# Are Quality-of-Life Scores Stable and Sensitive Over Time for Nursing Home Residents With and Without Dementia?

**DOI:** 10.1093/geroni/igaa057.181

**Published:** 2020-12-16

**Authors:** Tetyana Shippee, Xuanzi Qin, Zachary Baker, Stephanie Jarosek, Mark Woodhouse

**Affiliations:** University of Minnesota, Minneapolis, Minnesota, United States

## Abstract

The proportion of older adults with Alzheimer’s Disease and Related Dementias (ADRD) in nursing homes (NHs) has been increasing over time and creates a mandate to meaningfully examine their care. There is also a growing recognition that person-centered measures are important for dementia care, and consensus about the need to maximize residents’ quality of life (QoL). Yet, because QoL is fundamentally subjective, and residents with ADRD experience declines in cognitive function, their ability to make complex judgements about QoL has been questioned. This presentation will longitudinally assess whether QoL scores for residents with ADRD are stable and sensitive over time compared to those without ADRD. We use 2012-2015 Minnesota Resident Quality of Life and Satisfaction with Care Survey data, which contain in-person resident responses from a random sample of residents of all Medicare/Medicaid certified NHs in the state, about 40% of whom have AD/ADRD. These data were linked to the Minimum Data Set (MDS) 3.0. and facility characteristics data. The final sample contained 12,949 cohort-resident pairs, 8,803 unique residents, and 3,120 residents participated in more than two surveys. QoL scores of residents with and without ADRD were similarly stable over time and sensitive to health status change. We also found that stability of QoL scores may be driven by cognitive impairment as opposed to ADRD diagnoses. Thus, self-report QoL scores can also represent the QoL status for nursing home residents with ADRD diagnoses, and residents with ADRD diagnoses shouldn’t be excluded from quality of life surveys based on ADRD diagnoses.

